# Regional homogeneity alterations in multi-frequency bands in tension-type headache: a resting-state fMRI study

**DOI:** 10.1186/s10194-021-01341-4

**Published:** 2021-10-28

**Authors:** Shuxian Zhang, Huayun Li, Qinyan Xu, Chao Wang, Xue Li, Jiawei Sun, Yaqi Wang, Tong Sun, Qianqian Wang, Chengcheng Zhang, Jili Wang, Xize Jia, Xihe Sun

**Affiliations:** 1 Affiliated Hospital of Weifang Medical University, Weifang, Shandong Province China; 2 College of Teacher Education, Zhejiang Normal University, Jinhua, China; 3 Key Laboratory of Intelligent Education Technology and Application of Zhejiang Province, Zhejiang Normal University, Jinhua, China; 4 School of Information and Electronics Technology, Jiamusi University, Jiamusi, China; 5 Weifang Hospital of Traditional Chinese Medicine, Weifang, Shandong Province China; 6 Department of Medical Imaging, Weifang Medical University, Weifang, Shandong Province China; 7 Centre for Cognition and Brain disorders, the Affiliated Hospital of Hangzhou Normal University, Hangzhou, China

**Keywords:** Tension-type headache, Frequency-specific, Resting-state functional magnetic resonance imaging, Regional homogeneity (ReHo)

## Abstract

**Objectives:**

In this study, we aimed to investigate the spontaneous neural activity in the conventional frequency band (0.01−0.08 Hz) and two sub-frequency bands (slow-4: 0.027–0.073 Hz, and slow-5: 0.01–0.027 Hz) in tension-type headache (TTH) patients with regional homogeneity (ReHo) analyses.

**Methods:**

Thirty-eight TTH patients and thirty-eight healthy controls (HCs) underwent resting-state functional magnetic resonance imaging (RS-fMRI) scanning to investigate abnormal spontaneous neural activity using ReHo analysis in conventional frequency band (0.01−0.08 Hz) and two sub-frequency bands (slow-4: 0.027–0.073 Hz and slow-5: 0.01–0.027 Hz).

**Results:**

In comparison with the HC group, patients with TTH exhibited ReHo increases in the right medial superior frontal gyrus in the conventional frequency band (0.01−0.08 Hz). The between group differences in the slow-5 band (0.01–0.027 Hz) highly resembled the differences in the conventional frequency band (0.01−0.08 Hz); even the voxels with increased ReHo were spatially more extensive, including the right medial superior frontal gyrus and the middle frontal gyrus. In contrast, no region showed significant between-group differences in the slow-4 band (0.027–0.073 Hz). The correlation analyses showed no correlation between the ReHo values in TTH patients and VAS scores, course of disease and number of seizures per month in conventional band (0.01−0.08 Hz), slow-4 band (0.027–0.073 Hz), as well as in slow-5 band (0.01–0.027 Hz).

**Conclusions:**

The results showed that the superior frontal gyrus and middle frontal gyrus were involved in the integration and processing of pain signals. In addition, the abnormal spontaneous neural activity in TTH patients was frequency-specific. Namely, slow-5 band (0.01–0.027 Hz) might contain additional useful information in comparison to slow-4 band (0.027−0.073 Hz). This preliminary exploration might provide an objective imaging basis for the understanding of the pathophysiological mechanism of TTH.

**Supplementary Information:**

The online version contains supplementary material available at 10.1186/s10194-021-01341-4.

## Introduction

Tension-type headache (TTH) is the most common form of all headache disorders throughout the world [1]. In the Global Burden of Diseases, Injuries, and Risk Factors Study 2016 (GBD 2016), TTH ranks third in terms of global prevalence, second only to dental caries and latent tuberculosis infection [2]. As a major global public health concern, TTH has caused a significant impact on society as a whole, as well as on our daily lives. For example, the estimated total indirect annual financial losses to society due to TTH were CNY 233.2 billion [3] and Years Lived with Disability (YLDs) caused by TTHs were 7.2 million [2]. It has therefore become increasingly important to understand the pathophysiology of TTH, to develop an effective therapeutic agenda. Nevertheless, the exact mechanisms of TTH are not fully understood [4].

Evidence from the nociceptive flexion reflex data [5, 6] suggests that TTH is a disorder of an endogenous anti-nociceptive system, with a lowering of tone and recruitment of the descending pain modulatory system (DPMS) [7]. By structures such as the frontal cortex, rostral anterior cingulate gyrus, hypothalamus, insulae, periaqueductal grey (PAG), rostral ventromedial medulla, spinal cord, the DPMS produce an effect on pain perception, which is mediated by descending monoaminergic pathways utilizing serotonin or norepinephrine [8, 9]. Serotonin and norepinephrine modulate the perception of pain by regulating pain signals that come from the brain and travel down the spinal cord [8, 10]. In addition, Wang et al. [11] and Schmidt-Wilcke et al. [12] reported many abnormal brain functions and structures in TTH patients, including the middle frontal gyrus and the superior frontal gyrus, which represents the vast majority of the frontal cortex. Taken together, these studies emphasized the role of the DPMS in the occurrence and development of TTH.

Resting-state functional magnetic resonance imaging (RS-fMRI) is a noninvasive technology suitable for clinical conditions, which involves the study of spontaneous brain activity by detecting alterations related to blood oxygen level-dependent signals (BOLD) in individuals with no specific task or stimulation [13–15]. It was reported that characteristics of altered resting state may serve as useful markers to reflect the progress of a disease, which would be beneficial in understanding disease states [13, 14]. As a promising index of intrinsic brain activation, reported in 2004, regional homogeneity (ReHo) uses Kendall’s coefficient of concordance to measure regional synchronizations of the BOLD signal among neighboring voxels of the brain [15], to characterize the local neural activity in resting state. It was suggested that ReHo was a highly test-retest reliable characteristic of the human brain connectome [16], and it has neurobiological relevance probably determined by anatomical, developmental, and neurocognitive factors [16, 17]. Thus, ReHo has been suggested to serve as a potential neuroimaging marker for tracing the changes of functional homogeneity, and may provide insight into the pathophysiology of brain disorders [18]. Presently, ReHo analysis has been increasingly used to detect abnormalities of regional functional synchronization and to reveal the neural mechanisms in neurological and psychiatric disorders, such as depression [19], schizophrenia [20], Alzheimer’s disease [21, 22], and headache disorders [23–25]. However, to the best of our knowledge, ReHo studies of TTH remain largely unknown, which has restricted our understanding of this type of headache disorder.

Most previous RS-fMRI studies have examined spontaneous brain activity in the conventional frequency band (0.01−0.08 Hz), as the oscillatory activity in this frequency band has been thought to reflect gray matter signals [13, 22, 26, 27], since the first study of resting state conducted by Biswal focused on this frequency range of 0.01−0.08 Hz [26]. However, the human brain is a complex biological system that can generate a large number of oscillatory waves, and neural signals within different frequency bands exhibit different properties and physiological functions [28, 29]. Studies have suggested that the conventional frequency oscillation (0.01−0.08 Hz) can be subdivided into slow-4 (0.027–0.073 Hz) and slow-5 (0.01–0.027 Hz) [28, 29]. Notably, many studies have indicated that slow-4 (0.027–0.073 Hz) and slow-5 (0.01–0.027 Hz) contribute differently to the conventional frequency oscillation [29–32], and frequency-specific changes have been shown in many brain disorders [32–35]. For example, Xue et al. [36] reported that major depressive disorder patients showed increased ReHo values in the medial prefrontal cortex in the slow-5 band (0.01–0.027 Hz). In addition, Zhang et al. [37] reported that ReHo features in the slow-4 band (0.027–0.073 Hz) showed better classification accuracy (89 %), when compared with conventional frequency band (0.01−0.08 Hz) and slow-5 band (0.01–0.027 Hz) in cirrhotic patients with clinical hepatic encephalopathy (HE). These studies suggested that the pattern of intrinsic brain activity was sensitive to specific frequency bands. It is therefore important to consider the effects of sub-frequency bands (slow-4: 0.027–0.073 Hz and slow-5: 0.01–0.027 Hz) when examining spontaneous neural activity in TTH in the conventional frequency band (0.01−0.08 Hz).

To address these issues, we used ReHo analyses to examine the synchronizations of local brain activity in resting states based on conventional frequency band (0.01−0.08 Hz) and two sub-frequency bands (slow-4: 0.027–0.073 Hz and slow-5: 0.01–0.027 Hz). Based on previous reports of chronic pain conditions [34, 38–44], this study hypothesized that the patterns of low frequency brain activities in some brain regions of the descending pain pathway might be altered in TTH patients, and that the alterations might be more sensitive in certain sub-frequency bands.

## Materials and methods

### Subjects

Thirty-eight patients meeting the International Classification of Headache Disorders 3rd Edition, beta version criteria (ICHD-3 beta) [45] for TTH, were recruited at the outpatient clinic of the Affiliated Hospital of Weifang Medical University from May 2018 to July 2019. All patients were diagnosed by two neurologists and were required to meet the following four headache characteristics: bilateral, mild-to-moderate intensity, non-pulsating, and not aggravated by routine physical activity. The demographic and clinical data including age, sex, education, attack frequency, disease duration, and scores for the Visual Analogue Scale (VAS), as well as the scores for Depression, Anxiety and Stress Scale (DASS) were also obtained during the interview. Thirty-eight healthy controls (HCs) with no history of headache were also recruited. The inclusion criteria for all subjects were: (1) Han ethnicity; (2) 18−60 years of age; (3) right-handed; (4) no history of neurological and psychiatric diseases; and (5) no MRI contraindications. Exclusion criteria were: (1) alcohol, nicotine, or drug abuse; (2) known history of hypertension, diabetes, anxiety, depression, serious neurological disorders, or psychiatric disorders; (3) suffering from other types of headache or chronic pain disorders; (4) intracranial lesions in previous MRI or computed tomography scans; (5) pregnancy or menstrual period in women; and (6) claustrophobia. The TTH patients were scanned during pain-free periods. Patients were considered in the pain-free phase according to the diagnoses of the neurologist and radiologist. According to the above inclusion criteria, a total of five patients were excluded from the TTH group. Seven HCs were excluded due to large head motion (more than 3.0 mm of maximal translation and 3.0°of maximal rotation). So, 33 TTH patients and 31 HCs were enrolled in our study. The disease duration was 1.73 ± 1.14 years with TTH and frequency of 15.36 ± 3.89 attacks per month. Headache pain was reported bilateral, with a headache intensity of 4.84 ± 1.25 on a VAS score of 0-10 (See Table S1 in the supplementary materials for details). This study was approved by the Affiliated Hospital of Weifang Medical University Committee on Human Research. All TTH patients and healthy controls signed informed consent forms approved by the committee.

### MRI acquisition

A 3.0 T MRI scanning system (Signa HDxt, GE Medical Systems, Waukesha, WI, USA) equipped with an eight channel phase array head coil was used to acquire all MRI data. The participants were secured in the scanner with the head cushioned by plastic foam pads to minimize movement and with two appropriately-sized earplugs to reduce scanner noise. All images were acquired parallel to the plane connecting the anterior and posterior commissure, and the scanning range was from the top of the skull to the skull base. First, T_2_-weighted images were performed to exclude the possibility of clinically silent lesions for all subjects. Prior to three-dimensional T_1_-weighted images, the resting-state functional images were acquired to avoid the influence of a long-time scanning on the brain activity of the subjects. The subjects were asked to remain still and relaxed, close their eyes, but not to sleep during the functional session. The scanning was terminated if the subject complained of any discomfort. The parameters were as follows: (1) resting state fMRI using echo planar imaging, repetition time (TR) = 2,000 ms, echo time (TE) = 30 ms, flip angle = 90°, slice thickness = 4.0 mm, matrix= 64 × 64, field of view (FOV) = 240 × 240 mm^2^, number of slices = 32, total volume = 200, the session lasted 400 s, (2) three-dimensional high-resolution T_1_-weighted images using the spoiled gradient recalled acquisition, TR = 7.8 ms, TE = 3.0 ms, flip angle = 15°, slice thickness = 1.0 mm, FOV = 256 × 256 mm², matrix = 256 × 256, number of slices = 188, and the session lasted 250 s.

### Data preprocessing

The original functional data were preprocessed using the toolkits of RESTplus, version 1.24 [46] (http://www.restfmri.net) implemented using a MATLAB 2020a platform (MathWorks, Natick, MA, USA). Considering that the old and new versions of MATLAB might be different, we also simultaneously compared the spatial distribution patterns of brain regions in 2014a and 2020a, with the compared results in the supplementary materials (Figure S1-Figure S3 in the supplementary materials). The steps were as follow: (1) discard the first 10 of the 200-time points for stabilization of the magnetic field, (2) a slice-timing correction for the acquisition delay between slices, (3) head motion correction, (4) normalization. First, an individual structural image was co-registered to the mean functional image and was segmented into tissue segmentations of structural images. Then, the Diffeomorphic Anatomical Registration Through Exponentiated Lie algebra (DARTEL) tool was used to compute the transformation from individual space to MNI space (resampling voxel size = 3 mm × 3 mm × 3 mm), (5) detrending was used to eliminate the influence on signal caused by thermal drift during long-time scanning, (6) regressing out of covariates including Friston-24 parameters [47], and (7) use of a temporal filter with a bandpass of conventional frequency band (0.01−0.08 Hz), slow-4 band (0.027–0.073 Hz), and slow-5 band (0.01–0.027 Hz). Seven subjects in the HC group were excluded for further analysis due to large head motion (more than 3.0 mm of maximal translation in any direction of x, y, or z or 3.0 of maximal rotation throughout the course of scanning). Concurrently, we compared the spatial patterns with the maximal translations exceeding 2.5 mm or rotations over 2.5 with the results given in the supplementary materials (Figure S4-Figure S6 in the supplementary materials).

### Regional homogeneity calculations

Kendall’s coefficient of concordance (KCC) was used to evaluate ReHo, which was performed using the toolkits of RESTplus, version 1.24 [46]. The ReHo value was acquired by calculating the KCC of the time course of every 27 nearest neighboring voxels [15]. The ReHo value of each voxel was then divided by the global mean ReHo of each individual for standardization purposes. Note that the spatial smoothing (full-width at half-maximum [FWHM] = 6 mm) was performed after ReHo calculation. At the same time, we smoothed the filtered fMRI data with a 4 mm FWHM Gaussian kernel, and comparison brain maps of 4 mm and 6 mm are provided in the supplementary materials (Figure S7-Figure S9 in the supplementary materials).

### Statistical analysis

Statistical analyses of functional images were performed with DPABI, version 4.0 [48] (http://rfmri.org/dpabi) and SPM12 (http://www.fil.ion.ucl.ac.uk/spm). To examine ReHo differences between the TTH and HC groups, a two sample *t*-test was conducted of the whole brain. Age and the mean frame-wise displacement (FD) [49] were included as nuisance covariates in group comparisons. To determine the effects of age, we also compared spatial distribution patterns of brain regions before and after age regressions, and included the compared results in the supplementary materials (See Figure S10-Figure S12 in the supplementary materials for detail). Multiple comparison correction was performed based on Threshold-Free Cluster Enhancement (TFCE) theory [50] with family-wise error (FWE) corrections (*P*_FWE_ < 0.01, permutations = 5,000). Finally, Pearson’s correlation analyses were conducted to determine correlations between the ReHo values of brain regions, which showed significant between-group differences and clinical features (VAS scores, number of seizures per month and course of disease) in the TTH group (*P* < 0.05 was considered significant). In addition, signals of brain regions showing significant differences were extracted and plotted as bar graphs at conventional frequency band (0.01−0.08 Hz), slow-4 band (0.027–0.073 Hz), and slow-5 band (0.01–0.027 Hz) (Figure S13-Figure S17).

## Results

### Demographic data of TTH patients and HCs

The demographic characteristics of TTH patients and HCs are shown in Table 1. There was a total of 38 TTH patients and 38 HCs in the study, of which five TTH and seven HC individuals were excluded because of older age and excessive head motions, respectively, so 33 TTH patients and 31 HC individuals were finally enrolled. There was no significant difference in demographic variables (age, sex, education, and FD) between the groups. Considering the influence of the participants’ characteristics, we conducted the correlation between anxiety scores, depression scores and ReHo map and found no significant correlation after the Gaussian Random Field theory (GRF, voxel *p* < 0.05, cluster *p* < 0.05). To provide more information, an uncorrected *p* value = 0.01 was performed. The correlation results between ReHo map and anxiety scores and depression scores were added in Table S2-Table S3 and Figure S19-Fig. 23 in the supplementary materials. In addition, the results excluded two patients (one with higher anxiety score and one with higher depression score) was compared with the results with no patients excluded and the similar spatial patterns showed the weak influence of the anxiety and depression scores (Figure S24-Figure S26 in the supplementary materials).

**Table.1:** Demographic characteristics of tension-type headache patients and healthy controls

Information	TTH	HC	*p*-value
Age (mean ± SD)	42.27 ± 12.26	36.87 ± 10.01	0.059
Sex (M/F)	13/20	14/17	0.641
Education (mean ± SD)	10.24 ± 3.28	11.03 ± 2.77	0.202
FD (Jenkinson) (mean ± SD)	0.09 ± 0.04	0.08 ± 0.03	0.304
VAS (mean ± SD)	4.84 ± 1.25		
Attack frequency (times/months)	15.36 ± 3.89		
Disease duration (years)	1.73 ± 1.14		

*TTH* tension-type headache, *HC* healthy control, *FD* framewise displacement, *VAS* Visual Analogue Scale

The ReHo values of different TTH type (chronic TTH (24), episodic TTH (9), mixed (33)) in all the five clusters are extracted (Figure S27-Figure S31 in the supplementary materials).

### ReHo analyses of different frequency bands

**Fig. 1:**
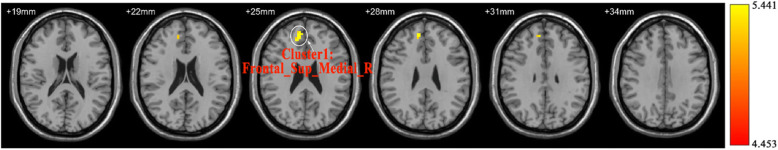
The regional homogeneity (ReHo) differences in the conventional band of 0.01–0.08 Hz. Warm colors indicate regions showing higher ReHo in TTH patients versus the HCs [*P*_FWE_ < 0.01, Threshold-Free Cluster Enhancement (TFCE) corrected]

In the conventional frequency band (0.01−0.08 Hz), only one cluster exhibited a significant increase relative to the HC group, with the peak coordinate located in the right medial superior frontal gyrus (Brodmann Area (BA) 32) (Table 2; Fig. 1).

**Table.2:** The regional homogeneity difference in each frequency band between tension-type headache patients and healthy controls

Cluster	Brain region			Cluster size	Coordinate (x, y, z)	Peak T-value	Effect size
	AAL	HCP	BA				
**Conventional frequency band (0.01-0.08 Hz)**							
**Cluster 1**	Right medial superior frontal gyrus	9m	32	21	9, 48, 24	5.4411	1.4687
**Slow-5 frequency band (0.01-0.027 Hz)**							
**Cluster 2**	Right medial superior frontal gyrus	9-46d	32	234	9, 45, 30	5.2924	1.4363
**Cluster 3**	Right middle frontal gyrus	46	46	39	36, 57, 24	4.0814	1.0425
**Cluster 4**	Right middle frontal gyrus	P9-46v	NAN	28	48, 33, 36	4.6041	1.1017
**Cluster 5**	Right middle frontal gyrus	8Av	9	17	39, 12, 51	3.9995	0.9786

Note: There is no significant cluster yielded in the slow-4 frequency band (0.027-0.073 Hz)

The clusters located in the white matter are not reported

We also provided the cluster names in other three functional templates (See Table S7 for details)

*Abbreviations*: *AAL *Anatomical Automatic Labeling, *HCP *Human Connectome Parcellation, *BA *Brodmann Area

**Fig. 2:**
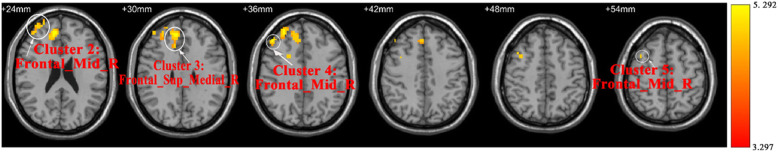
The regional homogeneity (ReHo) differences in the slow-5 band of 0.01–0.027 Hz. Warm colors indicate regions showing higher ReHo in TTH patients versus the HCs [*P*_FWE_ < 0.01, Threshold-Free Cluster Enhancement (TFCE) corrected]

In the slow-5 band (0.01–0.027 Hz), there were four clusters exhibiting significant increases relative to the HC group, with the peak coordinate located in the right medial superior frontal gyrus (Cluster 2, BA32; Considering the Cluster 2 spanning several brain regions of AAL, we showed the specific brain regions contained in the cluster in Figure S18 in the supplementary materials) and right middle frontal gyrus (Cluster 3, BA46; Cluster 4; Cluster 5, BA9) (Table 2; Fig. 2).

In the slow-4 band (0.027–0.073 Hz), no regions showed significant between-group differences between the TTH and HC groups.

### Correlation analyses

The regions showing significant between-group differences were extracted as regions of interest (ROIs), and five ROIs were defined for correlation analyses. The correlation analyses showed no correlation between the ReHo values in TTH patients and VAS scores, number of seizures per month and course of disease in conventional band (0.01−0.08 Hz), slow-4 band (0.027–0.073 Hz), as well as in slow-5 band (0.01–0.027 Hz). (See Table S4, Table S5, Table S6 in the supplementary materials for specific results of correlation analysis).

## Discussion

In this study, we used the ReHo method to investigate the spontaneous neural activities of TTH patients during resting states in the conventional frequency band (0.01−0.08 Hz) and two sub-frequency bands (slow-4: 0.027–0.073 Hz, and slow-5: 0.01–0.027 Hz). Our results showed that in the conventional frequency band (0.01−0.08 Hz), TTH patients exhibited increased ReHo in the medial superior frontal gyrus (BA32). The between-group differences in the slow-5 band (0.01–0.027 Hz) highly resembled the differences in the conventional frequency band (0.01−0.08 Hz), but the voxels with increased ReHo were spatially more extensive. In contrast, ReHo values in slow-4 band (0.027–0.073 Hz) analyses showed no significant group difference. Additionally, this study found no correlation between the ReHo values of the five significant group-different regions and VAS scores, course of disease and number of seizures per month in TTH patients in the conventional band (0.01−0.08 Hz), slow-4 band (0.027–0.073 Hz), as well as in slow-5 band (0.01–0.027 Hz). Our results therefore showed the benefits of sub-frequency band analyses and frequency-specific characteristic of ReHo changes in TTH patients.

The increased ReHo values of the medial superior frontal gyrus (BA32) and middle frontal gyrus (BA46, BA9) indicated increased local synchronization of low frequency fluctuations of the BOLD signal. As vital parts of the frontal lobe, the superior frontal gyrus and middle frontal gyrus participate in DPMS by modulating the cortical and subcortical damaging pathways during pain processing [51], which is dependent on its connections to other areas of the cerebral neocortex, hippocampus, PAG, thalamus, amygdala, and basal nuclei. Specifically, the medial superior frontal gyrus (BA32) belongs to the medial prefrontal cortex, which mediates antinociceptive effects, because it is the main source of cortical afferents to the PAG for the modulation of pain [51]. In addition, the middle frontal gyrus (BA46) and middle frontal gyrus (BA9) are two important components of the dorsolateral prefrontal cortex [52], considered to be a key node of networks implicated in nociceptive processing and pain modulation [53], which is involved in pain suppression along with cognitive and emotional control as well as pain detection. A study of Idiopathic Trigeminal Neuralgia (ITN) [54] showed that ReHo values of the superior frontal gyrus were significantly higher in ITN patients, when compared with HCs. In addition, a recent meta-analysis showed that anodal transcranial direct current stimulation of the dorsolateral prefrontal cortex reduced pain intensity in chronic pain patients [55]. Based on these results, our results of increased brain activity in the frontal lobe were consistent with previous studies on pain disorders [54, 55]. Thus, it is possible that the increased brain activity of the frontal lobe could raise the capacity for pain perception and modulation in TTH patients.

In contrast to an earlier fMRI study of TTH patients [11], which reported decreased ReHo values in the superior frontal gyrus and the middle frontal gyrus, the present study showed conflicting findings of increased ReHo values in these regions in TTH patients. Possible explanations concerning this discrepancy might be the strict TTH criteria employed in this study and different pathological stages of TTH patients.

The most important finding of the present study was that the abnormal spontaneous neural activity measured by RS-fMRI in TTH patients was frequency-specific. Specifically, ReHo changes were identified in the middle frontal gyrus and medial superior frontal gyrus in the slow-5 band (0.01–0.027 Hz), whereas no regions were identified in the slow-4 band (0.027−0.073 Hz). It is suggested that gray matter-related oscillations primarily occurred in slow-4 (0.027−0.073 Hz) and slow-5 (0.01–0.027 Hz) bands [29], and previous studies have shown that slow-4 (0.027−0.073 Hz) and slow-5 (0.01–0.027 Hz) bands had different levels of sensitivity to different brain disorders. For example, Meylakh et al. [56] reported that the PAG and hypothalamus displayed greater power in the slow-4 band (0.027–0.073 Hz) in the phase immediately prior to migraines, while Han et al. [30] suggested that slow-5 band (0.01–0.027 Hz) could be more sensitive in detecting abnormalities of spontaneous brain activities in mild cognitive impairment patients. In the present study, our results displayed that the slow-5 band (0.01–0.027 Hz) was more sensitive in detecting ReHo abnormalities in TTH patients, when compared to slow-4 band (0.027−0.073 Hz), suggesting that ReHo analysis of slow-5 band (0.01–0.027 Hz) may provide additional useful information, when compared with slow-4 band (0.027−0.073 Hz). However, whether such frequency-specific fluctuations can be used for the diagnosis of TTH remains to be further investigated, while other frequency-dependent and frequency-independent characteristics of brain networks need further investigation.

The effect of age on resting-state brain function has been extensively studied, and age-related changes have been reported in many investigations [57–59]. Considering the possible influence of age, we regressed the age in the two sample *t*-test, and spatial patterns of results before and after regression were compared. The results showed that although there were slight differences (right superior occipital gyrus), the spatial distribution patterns were generally similar, which indicated that age had a weak impact on the results. Superior occipital gyrus is a part of the occipital lobe, which is the center of the visual cortex, responsible for the reception and processing of visual information [60], but the role of the occipital lobe in pain processing has not yet been reported, and therefore needs further investigation.

Pain is an unpleasant subjective feeling and emotional experience, and transmission of pain in the central nervous system is highly complex, involving multiple brain regions. As the most fully described pain modulatory circuit, the descending pain modulatory system (DPMS) regulates nociceptive processing in favor of facilitation or inhibition, which is related to the pain experience in different situations [8, 9]. The frontal lobe and periaqueductal gray (PAG) are the critical components of the DPMS [61]. The PAG receives input from higher cortical sites including the frontal lobe and insula, and has reciprocal connections with the amygdala and ascending input from the spinal cord using the parabrachial nuclei [61–64]. The current study showed higher ReHo values in the frontal lobe, which indicated functional compensation. The importance of the frontal lobe in planning complex behavior has been previously reported [65]. Functional neuroimaging studies have shown that attention, emotion, and expectation of pain modulated PAG activation [66–68], and functional compensation in the frontal lobe involved dysfunctions of the PAG.

We tried our best to enroll as much patients as possible. However, the fMRI data of the TTH patients are rare. Wang et al. [11] conducted the first and the only published fMRI study on the TTH, only 10 patients were enrolled. In addition, the pain is unbearable when headache occurs, so it is difficult for patients to perform fMRI scanning in this state. All the TTH patients were scanned during the headache-free period. Whether the two different periods showed differences in the tension type headache needs to be further studied.

Considering the complex condition of TTH, although the patients with anxiety disorder and depression disorder were excluded, the correlation analysis between ReHo map and anxiety scores and depression was performed. Neither the anxiety scores nor the depression scores were significantly correlated with the ReHo values. The similar conclusion was found in a study measuring the functional connectivity of the pain-free participants [69]. Due to the small sample size, we want to make further exploration and provide more information, an uncorrected *p* value = 0.01 was applied in the present study. Both anxiety and depression scores of the DASS showed positive correlation of ReHo values in the postcentral gyrus in the slow-5 frequency band of 0.01-0.027 Hz. Previous study has proposed that the postcentral gyrus plays an important role in emotional regulation [70]. The alteration of amplitude of low-frequency fluctuation (ALFF) and ReHo values in the postcentral gyrus could be used as a biomarker for generalized anxiety disorder [71]. Besides, Qi et al. [72] found increased gray matter volume in depression patients with anxiety disorder compared with healthy controls in the postcentral gyrus. Interestingly, the primary somatosensory cortex which is involved in perception of the intensity of pain is located in the postcentral gyrus [70, 73]. However, there is no group difference between TTH patients and healthy controls. The reason may be that the sample size is relatively small and the VAS scores of TTH patients enrolled in the current study is not high which represent a low pain intensity. In the further study, the sample size needs to be increased and the different pain levels should be taken into account.

Although our research was informative with respect to increased ReHo values in the conventional frequency band (0.01−0.08 Hz) as well as in the slow-5 band (0.01–0.027 Hz) in TTH patients, this study had several limitations. First, the sample size was relatively small, which might have some impact on the results. In this regard, it is important to increase the sample size and investigate different groups in future studies. To facilitate the meta-analysis and later studies, we shared the uncorrected *t* maps (http://www.restfmri.net/rsfmri/TTH.tar). Second, small head motion was unavoidable even though the subjects were instructed to remain still and relaxed. We therefore examined each image, and individuals with maximal translations exceeding 3.0 mm or rotations over 3.0° were excluded. In addition, FD was regressed to eliminate the influence of head movements as much as possible. Finally, our research was confined to TTH during headache-free periods. In future studies, further investigations of dysfunctions in TTH patients during the headache-ictal periods should be conducted.

## Conclusions

ReHo analysis was used to compare difference in resting-state brain functions between TTH and HC groups in the conventional frequency band (0.01−0.08 Hz) and two sub-frequency bands (slow-4: 0.027–0.073 Hz and slow-5: 0.01–0.027 Hz). The results showed that abnormal spontaneous neural activity in TTH patients was frequency specific. Specifically, slow-5 band (0.01–0.027 Hz) may contain additional useful information in comparison to slow-4 band (0.027−0.073 Hz). This preliminary study provided an objective imaging basis for the pathophysiological mechanism of TTH. To the best of our knowledge, this is the first study to clarify ReHo abnormalities in sub-frequency bands in TTH patients, so our results increased our understanding of the pathophysiology responsible for TTH.

## Supplementary Information

**Additional file 1.**

## Data Availability

Raw data were generated at the Affiliated Hospital of Weifang Medical University. Derived data supporting the findings of this study are available from the corresponding author Xize Jia on request.

## Abbreviations

TTH: Tension-type headache
ReHo: Regional homogeneity
RS-fMRI: Resting-state functional magnetic resonance imaging
HCs: Healthy controls
GBD 2016: Global Burden of Diseases, Injuries, and Risk Factors Study 2016
YLDs: Years Lived with Disability
DPMS: Descending pain modulatory system
rACC: Rostral anterior cingulate gyrus
PAG: Periaqueductal grey
BOLD: Blood oxygenation level-dependent
HE: Hepatic encephalopathy
ICHD- 3 beta: *International Classification of Headache Disorders, Third Edition, Beta Version*
VAS: Visual Analogue Scale
TR: Repetition time
TE: Echo time
FOV: Field of view
DARTEL: Diffeomorphic anatomical registration through exponentiated Lie algebra
KCC: Kendall’s coefficient of concordance
FWHM: Full-width at half-maximum
FD: Frame-wise displacement
TFCE: Threshold-Free Cluster Enhancement
FWE: Family-wise error
ROIs: Regions of interest
BA: Brodmann Area
ITN: Idiopathic trigeminal neuralgia
