# Supplementary material for: Regional homogeneity alterations in multi-frequency bands in tension-type headache: a resting-state fMRI study
Source: J Headache Pain. 2021 Oct 28;22(1):129. doi: 10.1186/s10194-021-01341-4 (PMC8555254; doi:10.1186/s10194-021-01341-4)
Supplement: Supplementary file 1 — Additional file 1. [file 10194_2021_1341_MOESM1_ESM.docx]

**Regional homogeneity alterations in multi-frequency bands in tension-type headache:** **a resting-state fMRI Study**


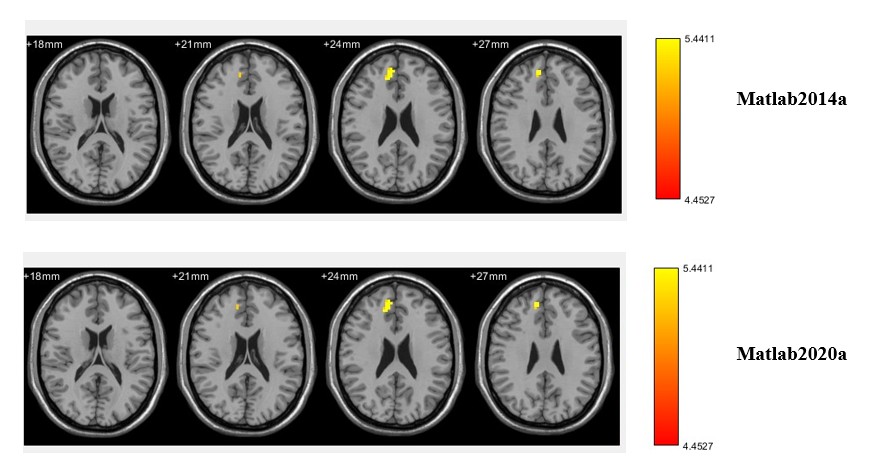


**Figure S1.** Patterns of results that analyzed with Matlab 2014a (Top) and patterns of results that analyzed with Matlab 2020a (Bottom) in the conventional frequency band (0.01-0.08 Hz).


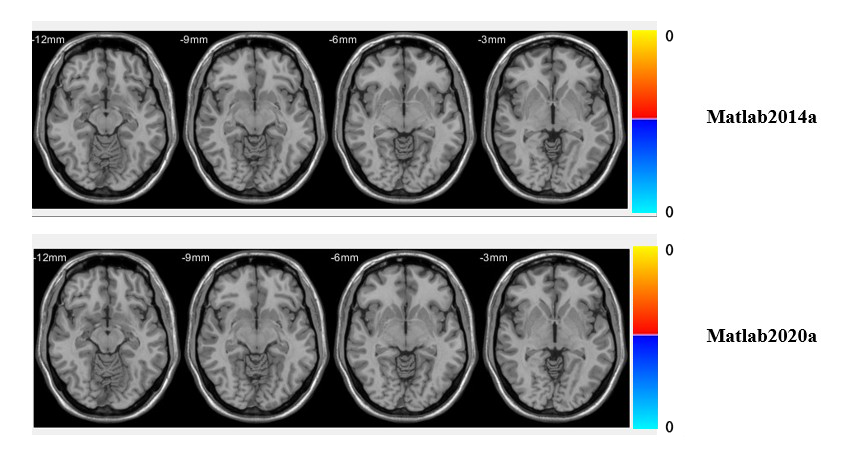


**Figure S2.** Patterns of results that analyzed with Matlab 2014a (Top) and patterns of results that analyzed with Matlab 2020a (Bottom) in the slow-4 frequency band (0.027-0.073 Hz).


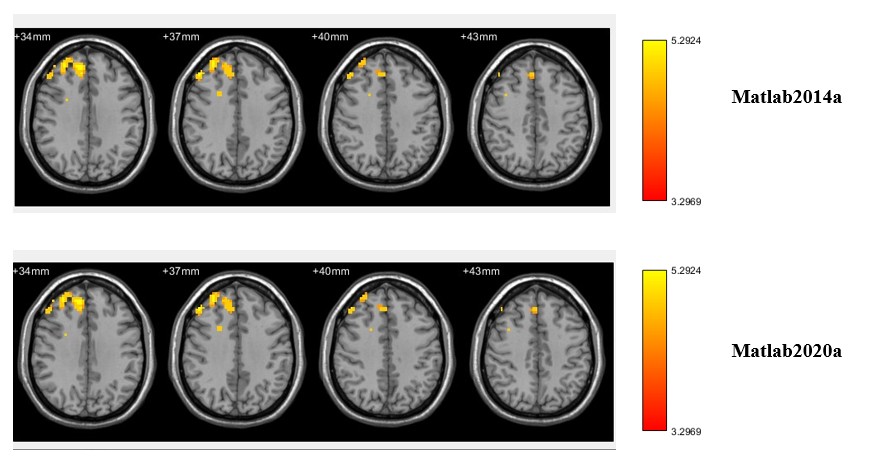


**Figure S3.** Patterns of results that analyzed with Matlab 2014a (Top) and patterns of results that analyzed with Matlab 2020a (Bottom) in the slow-5 frequency band (0.01-0.027 Hz).


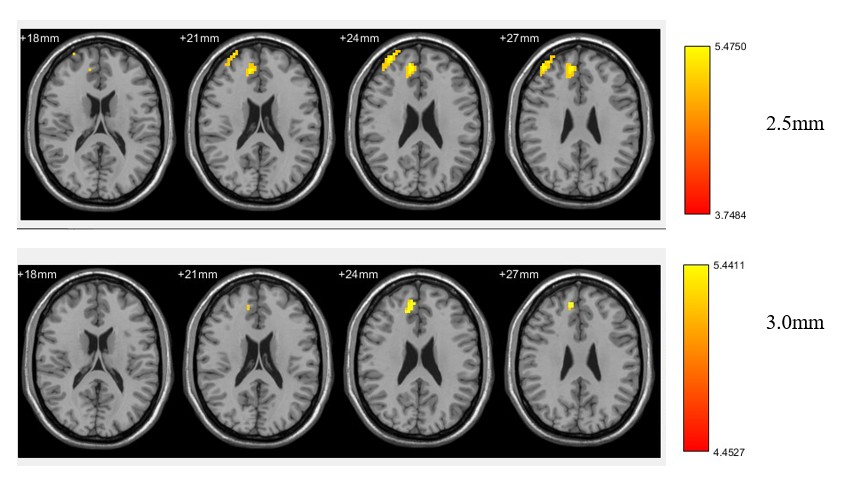


**Figure S4.** Patterns of results that analyzed with head motion threshold of 2.5 mm or 2.5° (Top) and patterns of results that analyzed with head motion threshold of 3.0 mm or 3.0°(Bottom) in the conventional frequency band (0.01-0.08 Hz).


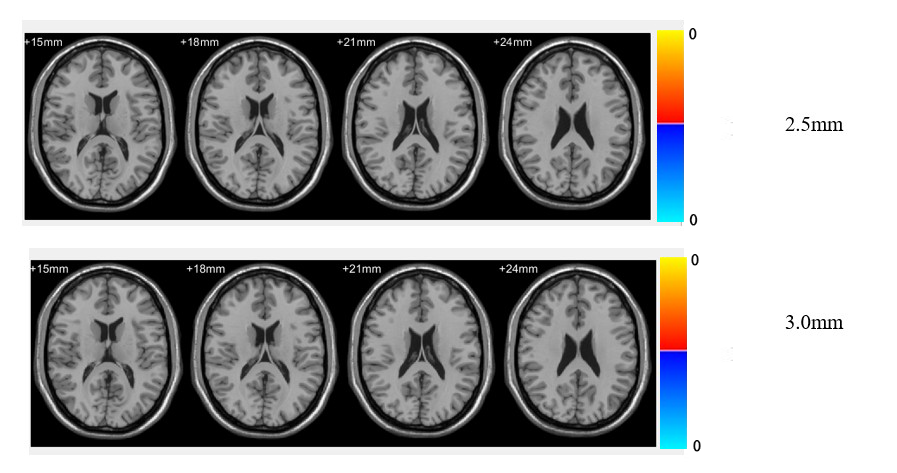


**Figure S5.** Patterns of results that analyzed with head motion threshold of 2.5 mm or 2.5° (Top) and patterns of results that analyzed with head motion threshold of 3.0 mm or 3.0°(Bottom) in the slow-4 frequency band (0.027-0.073 Hz).


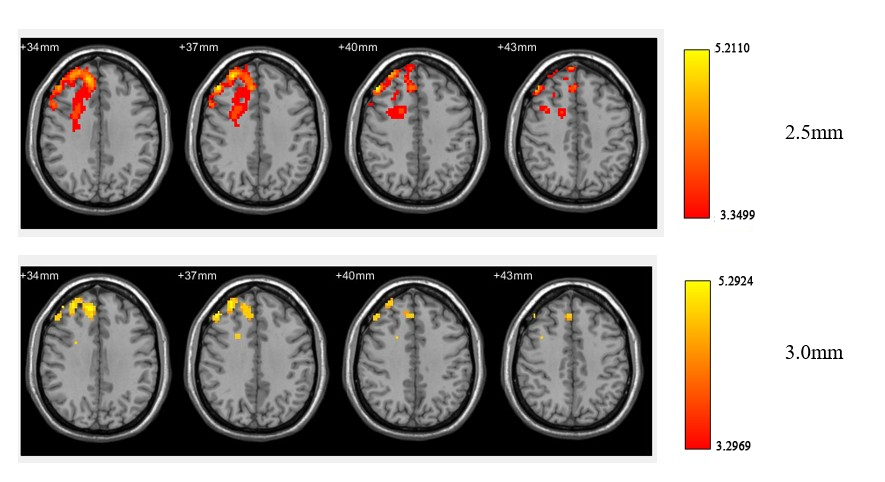


**Figure S6.** Patterns of results that analyzed with head motion threshold of 2.5 mm or 2.5° (Top) and patterns of results that analyzed with head motion threshold of 3.0 mm or 3.0°(Bottom) in the slow-5 frequency band (0.01-0.027 Hz).


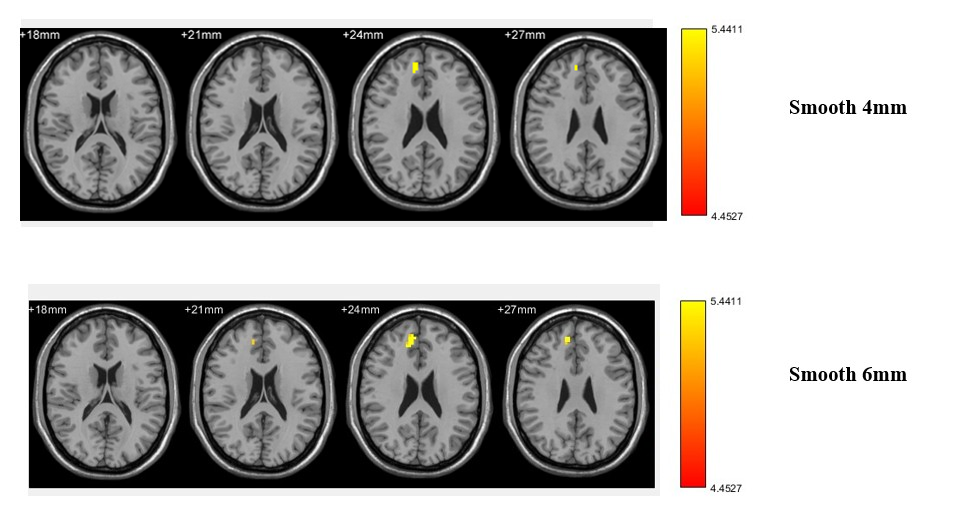


**Figure S7.** Patterns of results that analyzed with 4 mm smooth kernel (Top) and patterns of results that analyzed with 6 mm smooth kernel (Bottom) in the conventional frequency band (0.01-0.08 Hz).


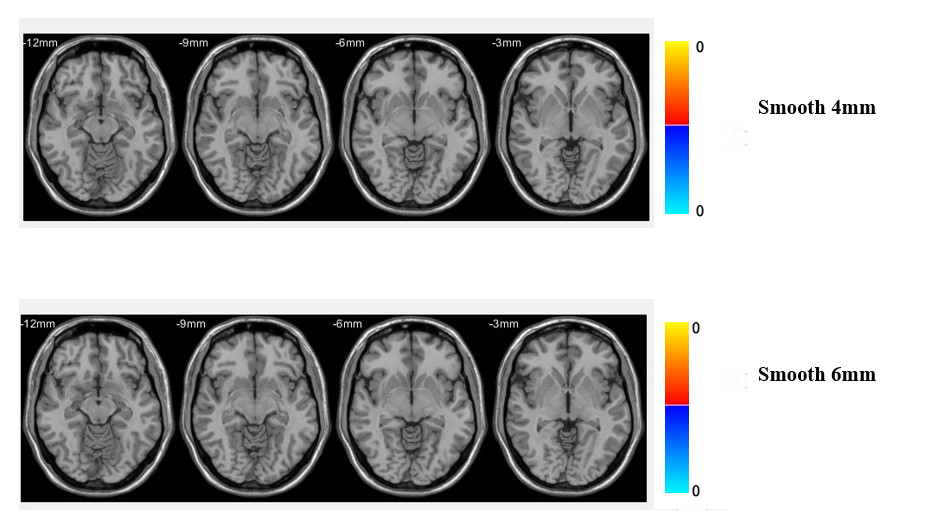


**Figure S8.** Patterns of results that analyzed with 4 mm smooth kernel (Top) and patterns of results that analyzed with 6 mm smooth kernel (Bottom) in the slow-4 frequency band (0.027-0.073 Hz).


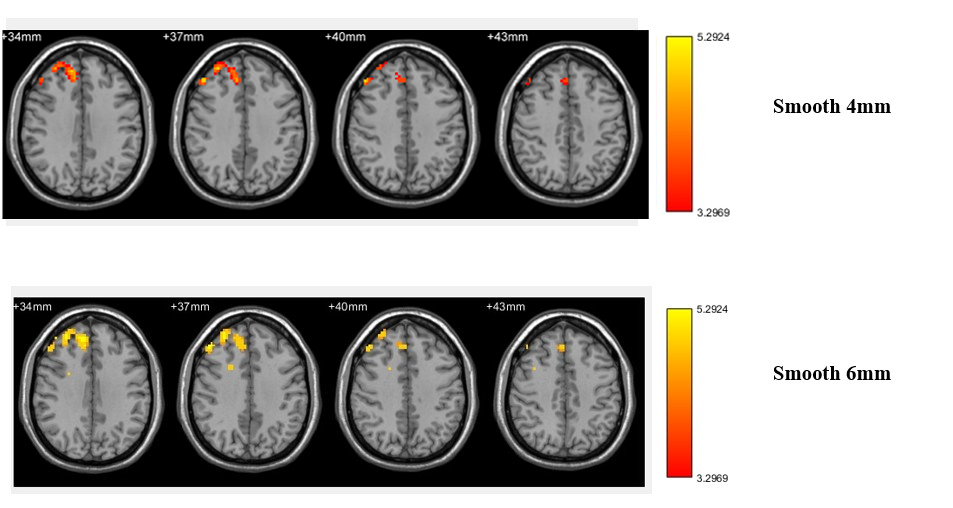


**Figure S9.** Patterns of results that analyzed with 4 mm smooth kernel (Top) and patterns of results that analyzed with 6 mm smooth kernel (Bottom) in the slow-5 frequency band (0.01-0.027 Hz).


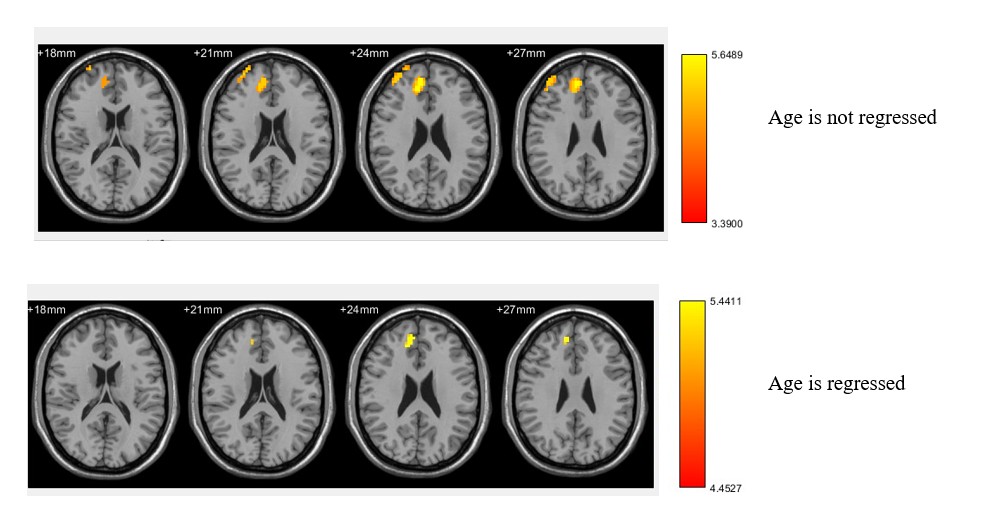


**Figure S10.** Patterns of results that age is not regressed (Top) and patterns of results that age is regressed (Bottom) in the conventional frequency band (0.01-0.08 Hz).


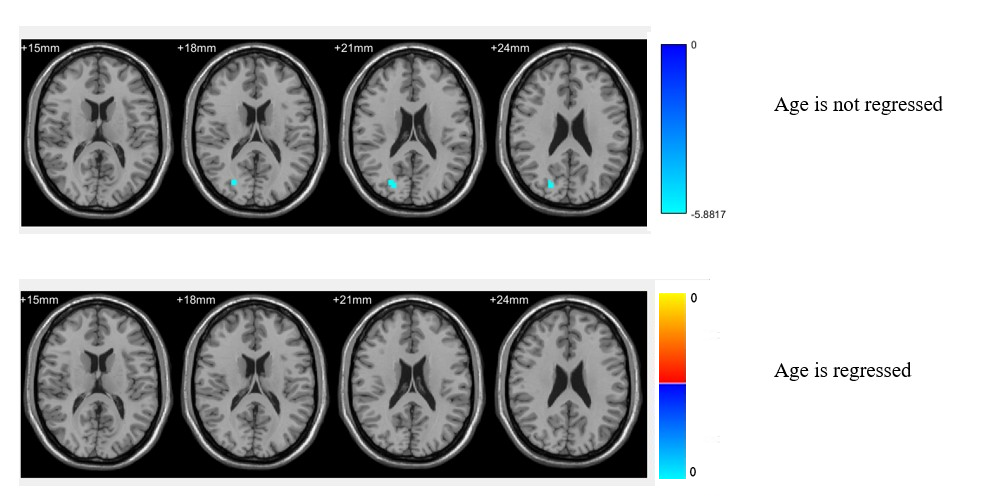


**Figure S11.** Patterns of results that age is not regressed (Top) and patterns of results that age is regressed (Bottom) in the slow-4 frequency band (0.027-0.073 Hz).


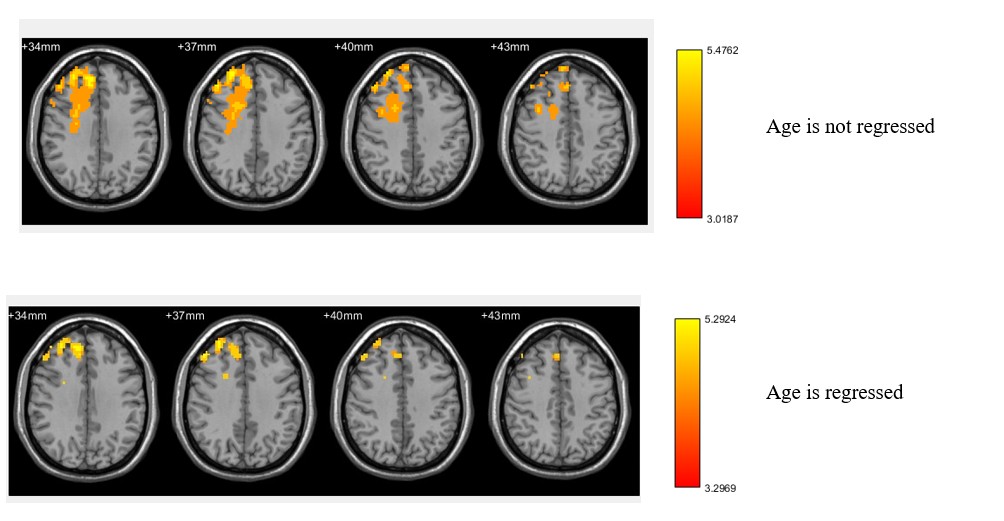


**Figure S12.** Patterns of results that age is not regressed (Top) and patterns of results that age is regressed (Bottom) in slow-5 frequency band (0.01-0.027 Hz).


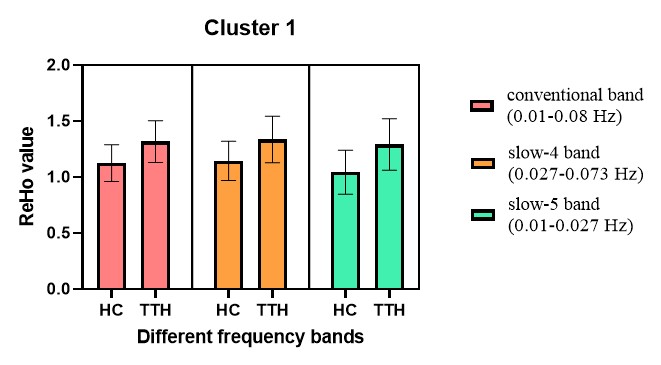


**Figures S13.** ReHo values in all the three frequency bands were extracted using the Cluster 1 (Right medial superior frontal gyrus, MNI coordinate: 9, 48, 24).


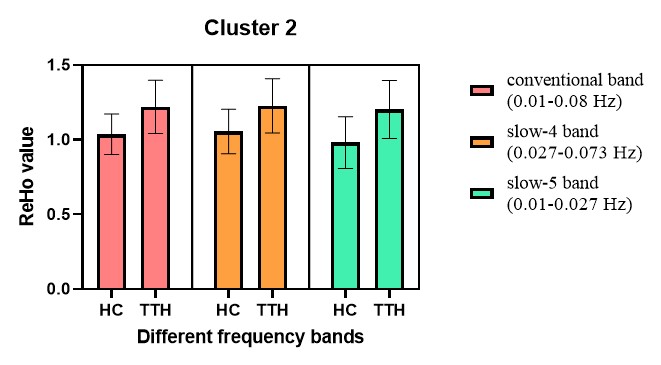


**Figures S14.** ReHo values in all the three frequency bands were extracted using the Cluster 2 (Right medial superior frontal gyrus, MNI coordinate: 9, 45, 30).


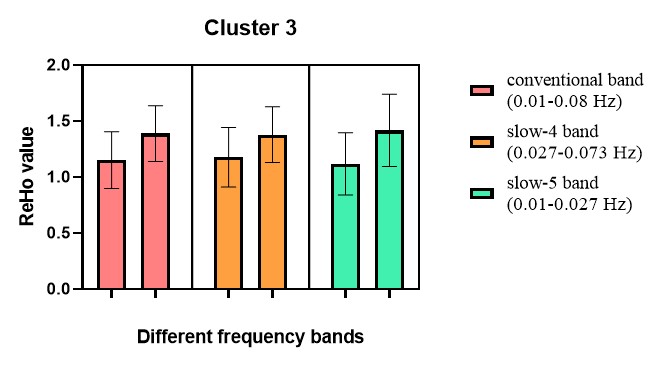


**Figures S15.** ReHo values in all the three frequency bands were extracted using the Cluster 3 (Right medial frontal gyrus, MNI coordinate: 36, 57, 24).


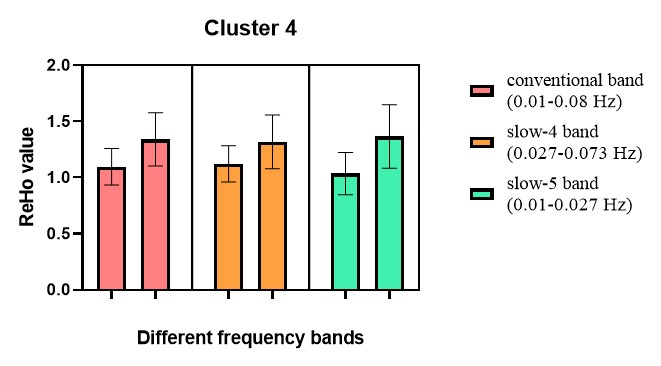


**Figures S16.** ReHo values in all the three frequency bands were extracted using the Cluster 4 (Right medial frontal gyrus, MNI coordinate: 48, 33, 36).


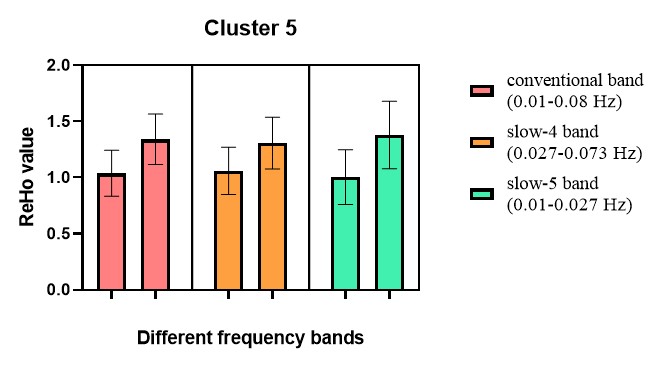


**Figures S17.** ReHo values in all the three frequency bands were extracted using the Cluster 5 (Right medial frontal gyrus, MNI coordinate: 39, 12, 51).


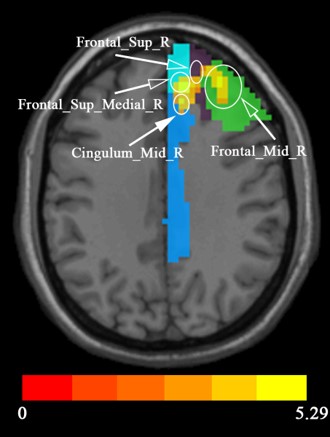


**Figure S18.** The main brain regions of the Cluster 2 (MNI coordinate: 9, 45, 30) which spans four brain regions of AAL template. The Frontal_Sup_Medial_R is the region we reported in the Table 2 in the manuscript due to its maximum t value. The other three regions are the sub regions within the Cluster 2.

**Note:** The underlay is Ch2 template. The light blue area is the Frontal_Sup_R region in the AAL template. The dark blue area is the Frontal_Sup_Medial_R region in the AAL template. The purple area is the Frontal_Sup_R region in the AAL template. The green area is the Frontal_Mid_R region in the AAL template.

**Abbreviations:** Frontal_Sup_R = Right superior frontal gyrus; Frontal_Sup_Medial_R = Right medial superior frontal gyrus, medial; Cingulum_Mid_R = Right median cingulate and paracingulate gyri; Frontal_Mid_R = Right middle frontal gyrus

**
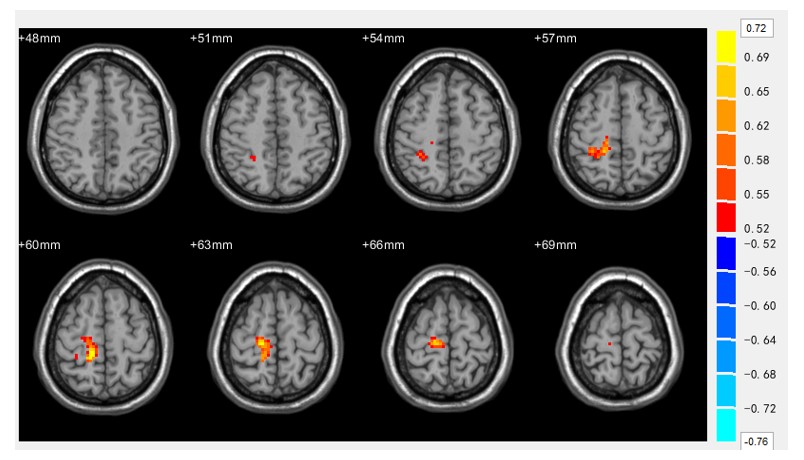
**

**Figure S19.** Correlation between ReHo map and anxiety scores of DASS in the conventional frequency band (0.01-0.08 Hz) (Uncorrected *p* < 0.01, cluster size = 50 voxels).

**
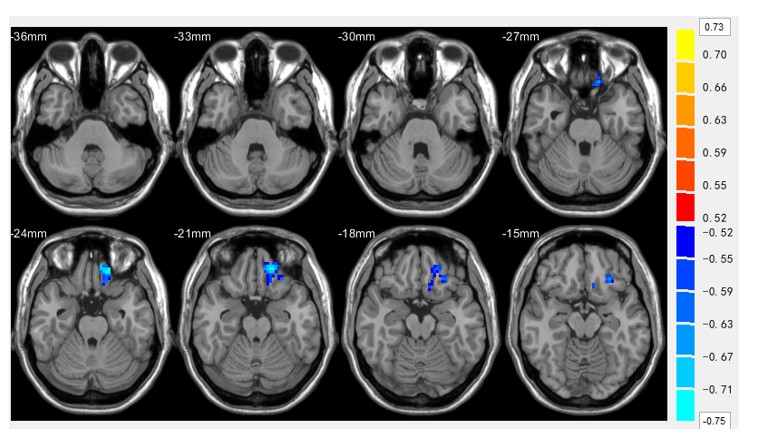
**

**Figure S20.** Correlation between ReHo map and anxiety scores of DASS in the slow-4 frequency band (0.027-0.073 Hz) (Uncorrected *p* < 0.01, cluster size = 50 voxels).


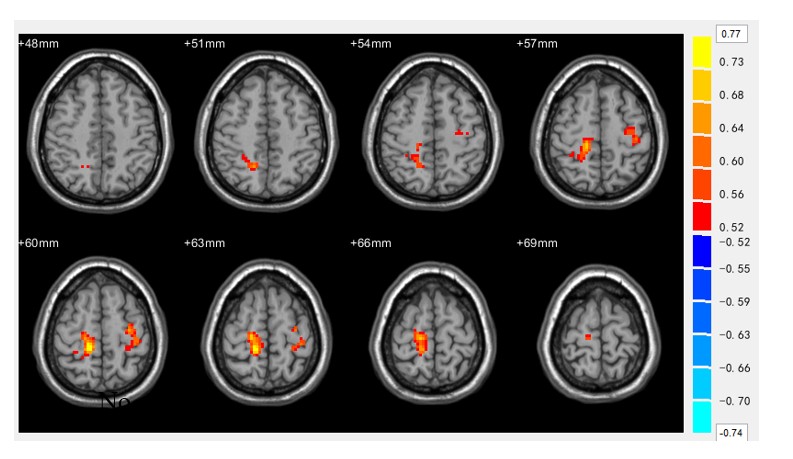


**Figure S21.** Correlation between ReHo map and anxiety scores of DASS in the slow-5 frequency band (0.01-0.027 Hz) (Uncorrected *p* < 0.01, cluster size = 50 voxels).

**
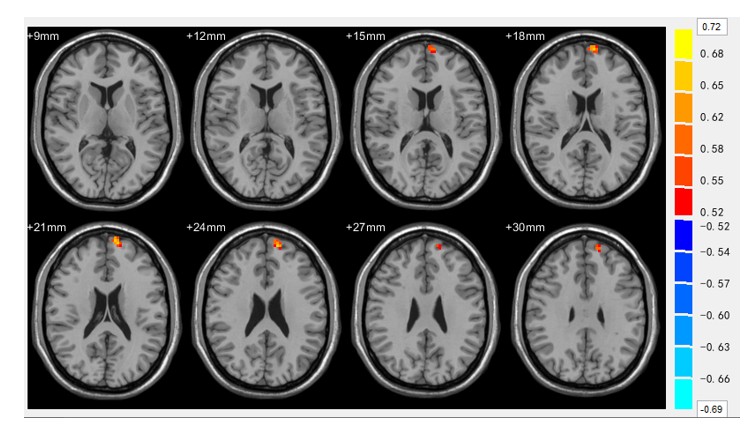
**

**Figure S22.** Correlation between ReHo map and depression scores of DASS in the slow-4 frequency band (0.027-0.073 Hz) (Uncorrected *p* < 0.01, cluster size = 50 voxels).


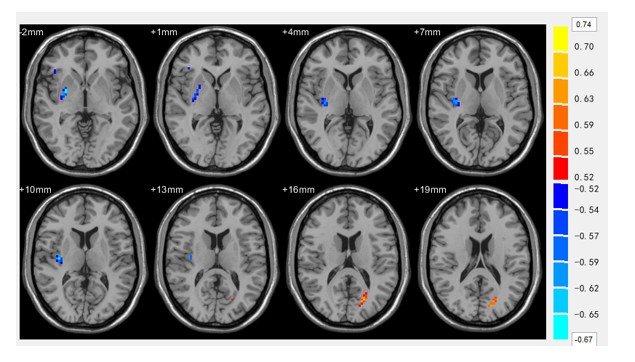


**Figure S23.** Correlation between ReHo map and depression scores of DASS in the slow-5 frequency band (0.01-0.027 Hz) (Uncorrected *p* < 0.01, cluster size = 50 voxels).


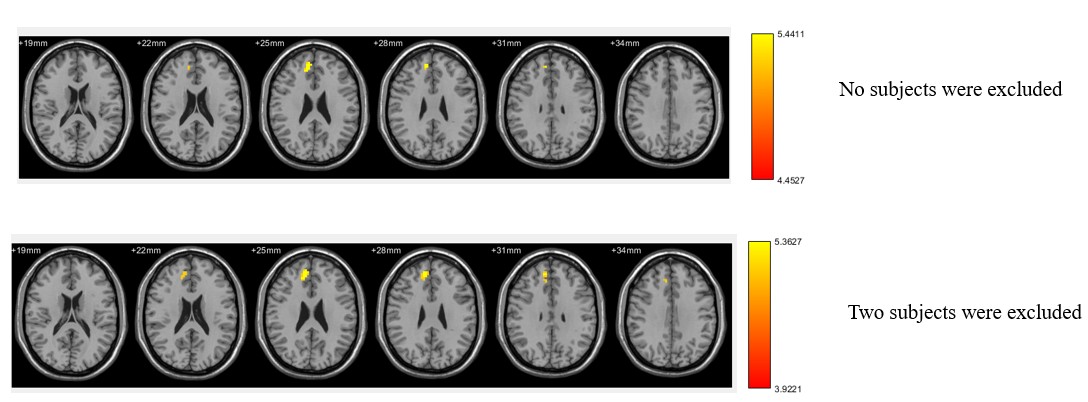


**Figure S24.** Patterns of results that no patients were excluded (Top) and patterns of results that two patients were excluded (Bottom) (one with higher anxiety score and one with higher depression score) in conventional frequency band (0.01-0.08 Hz).

**
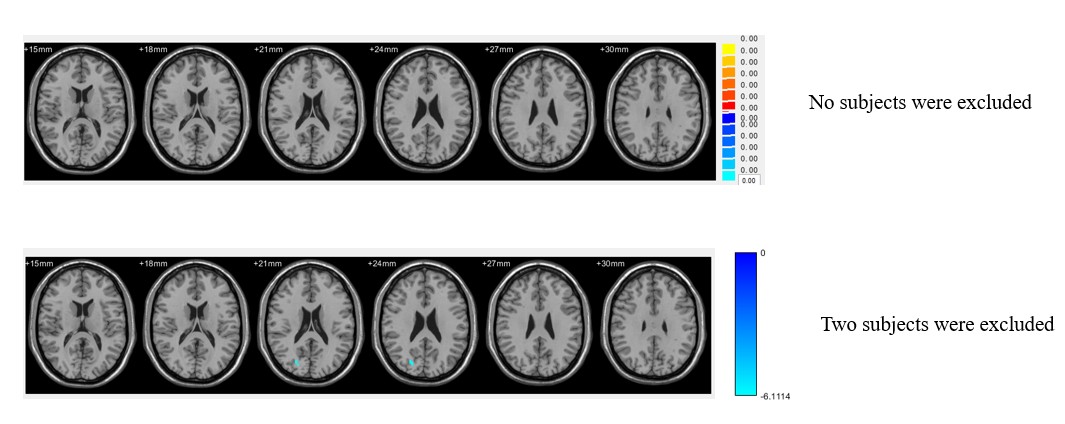
Figure S25.** Patterns of results that no patients were excluded (Top) and patterns of results that two patients were excluded (Bottom) (one with higher anxiety score and one with higher depression score) in slow-4 frequency band (0.027-0.072 Hz).

**
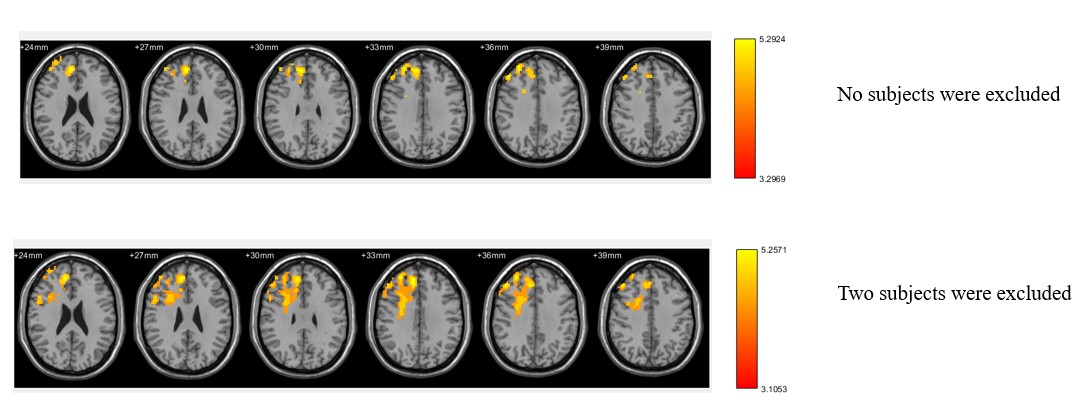
**

**Figure S26.** Patterns of results that no patients were excluded (Top) and patterns of results that two patients were excluded (Bottom) (one with higher anxiety score and one with higher depression score) in slow-5 frequency band (0.027-0.073 Hz).

**
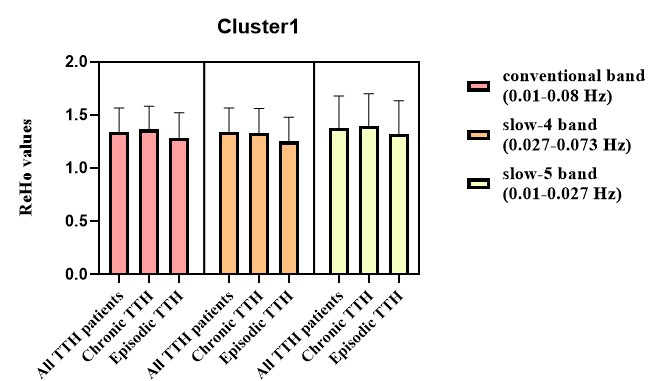
**

**Figure S27.** ReHo values of different TTH type (chronic TTH (24), episodic TTH (9), mixed (33)) in the Cluster 1.

**
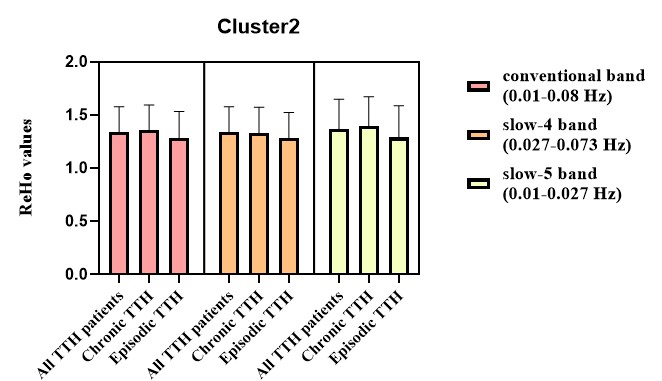
**

**Figure S28.** ReHo values of different TTH type (chronic TTH (24), episodic TTH (9), mixed (33)) in the Cluster 2.

**
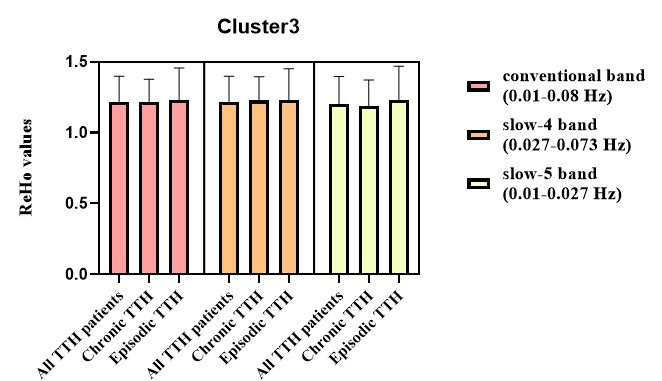
**

**Figure S29.** ReHo values of different TTH type (chronic TTH (24), episodic TTH (9), mixed (33)) in the Cluster 3.

**
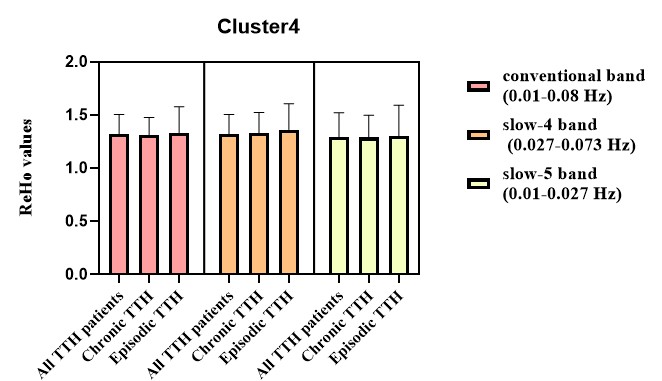
**

**Figure S30.** ReHo values of different TTH type (chronic TTH (24), episodic TTH (9), mixed (33)) in the Cluster 4.

**
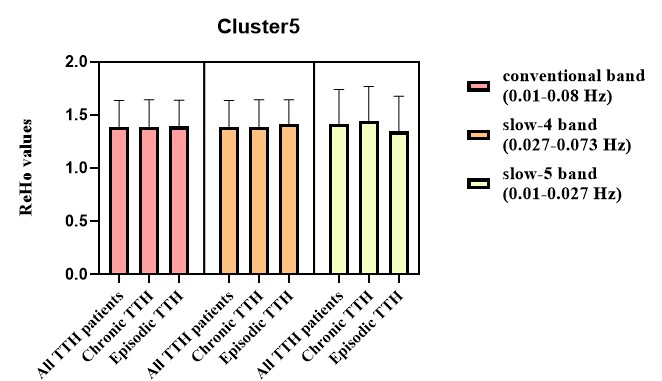
**

**Figure S31.** ReHo values of different TTH type (chronic TTH (24), episodic TTH (9), mixed (33)) in the Cluster 5.

**Table S1. Demographic characteristics of tension-type headache patients and healthy controls**

| **Information** | **TTH** | **HC** | ***P* value** |
| --- | --- | --- | --- |
| Age (mean ± SD) | 42.27 ± 12.26 | 36.87 ± 10.01 | 0.059 |
| Sex (M/F) | 13/20 | 14/17 | 0.641 |
| Education (mean ± SD) | 10.24 ± 3.28 | 11.03 ± 2.77 | 0.202 |
| FD (Jenkinson) (mean ± SD) | 0.09 ± 0.04 | 0.08 ± 0.03 | 0.304 |
| VAS (mean ± SD) | 4.84 ± 1.25 |  |  |
| Attack frequency (times/months) | 15.36 ± 3.89 |  |  |
| Disease duration (years) | 1.73 ± 1.14 |  |  |
| Anxiety scores of DASS | 3.33 ± 2.33 |  |  |
| Depression scores of DASS | 3.13 ± 2.88 |  |  |
| Stress scores of DASS | 5.42 ± 3.62 |  |  |

**Note:** The Anxiety, Depression, Stress scores of DASS scale are not complete, which only including 25 patients.

TTH: *tension-type headache,* HC: *healthy control,* FD: *framewise displacement*

VAS: *Visual Analogue Scale.*

**Table S2. Correlation between ReHo map and anxiety scores of DASS scale in all the three frequency bands**

| **Brain regions** | **Bormann area** | **Cluster size** | **Coordinate**  **(x, y, z)** | **Peak *r* value** |
| --- | --- | --- | --- | --- |
| **Conventional frequency band (0.01-0.08 Hz)** | | | | |
| Left superior frontal gyrus, orbital part | 11 | 111 | -18, 45, -21 | -0.76147 |
| Right postcentral gyrus | - | 120 | 15, -39,60 | 0.72338 |
| **Slow-4 frequency band (0.027-0.073 Hz)** | | | | |
| Left superior frontal gyrus, orbital part | 11 | 116 | -18, 39, -24 | -0.75003 |
| **Slow-5 frequency band (0.01-0.027 Hz)** | | | | |
| Left superior frontal gyrus, orbital part | 11 | 88 | -18, 45, -21 | -0.73853 |
| Right postcentral gyrus | - | 176 | 15, -33, 60 | 0.76834 |
| Left postcentral gyrus | 6 | 67 | -30, -15, 60 | 0.66778 |

**Table S3. Correlation between ReHo map and depression scores of DASS scale in all the three frequency bands**

| **Brain regions** | **Bormann area** | **Cluster size** | **Coordinate**  **(x, y, z)** | **Peak *r* value** |
| --- | --- | --- | --- | --- |
| **Slow-4 frequency band (0.027-0.073 Hz)** | | | | |
| Left superior frontal gyrus, medial | 10 | 54 | -9, 66, 21 | 0.67307 |
| **Slow-5 frequency band (0.01-0.027 Hz)** | | | | |
| Right inferior frontal gyrus, orbital part | 47 | 52 | 45, 24, -12 | -0.61778 |
| Right lenticular nucleus, pallidum | 48 | 53 | 27, -3, -3 | -0.6654 |
| Left superior occipital gyrus | 18 | 51 | -21, -72, 15 | 0.67398 |
| Left superior parietal gyrus | 7 | 65 | -21, -60, 48 | 0.72235 |
| Right postcentral gyrus | 4 | 84 | 15, -33, 63 | 0.73723 |

Note: No cluster was found in the conventional frequency band (0.01-0.08 Hz).

**Table S4. Correlation between ReHo values and VAS scores in all the three frequency bands**

| **Regions** |  | **VAS scores** |
| --- | --- | --- |
| **Conventional frequency band (0.01-0.08 Hz)** |  |  |
| Right medial superior gyrus | Pearson correlation (*r*) | 0.199 |
|  | Significance (*p*) | 0.266 |
| **Slow-5 band (0.01-0.027 Hz)** |  |  |
| Right medial superior frontal gyrus | Pearson correlation (*r*) | 0.243 |
|  | Significance (*p*) | 0.173 |
| Right middle frontal gyrus | Pearson correlation (*r*) | 0.219 |
|  | Significance (*p*) | 0.220 |
| Right middle frontal gyrus | Pearson correlation (*r*) | -0.172 |
|  | Significance (*p*) | 0.38 |
| Right middle frontal gyrus | Pearson correlation (*r*) | 0.289 |
|  | Significance (*p*) | 0.103 |

VAS: *Visual Analogue Scale.*

**Table S5. Correlation between ReHo values and number of seizures per month in all the three frequency bands**

| **Regions** |  | **Number of seizures per month** |
| --- | --- | --- |
| **Conventional frequency band (0.01-0.08 Hz)** |  |  |
| Right medial superior gyrus | Pearson correlation (*r*) | 0.047 |
|  | Significance (*p*) | 0.794 |
| **Slow-5 band (0.01-0.027 Hz)** |  | -0.069 |
| Right medial superior frontal gyrus | Pearson correlation (*r*) | 0.703 |
|  | Significance (*p*) | -0.069 |
| Right middle frontal gyrus | Pearson correlation (*r*) | 0.702 |
|  | Significance (*p*) | -0.069 |
| Right middle frontal gyrus | Pearson correlation (*r*) | -0.093 |
|  | Significance (*p*) | 0.608 |
| Right middle frontal gyrus | Pearson correlation (*r*) | -0.019 |
|  | Significance (*p*) | 0.914 |

**Table S6. Correlation between ReHo values and course of duration in all the three frequency bands**

| **Regions** |  | **Disease duration** |
| --- | --- | --- |
| **Conventional frequency band (0.01-0.08 Hz)** |  |  |
| Right superior gyrus, medial | Pearson correlation (*r*) | 0.032 |
|  | Significance (*p*) | 0.861 |
| **Slow-5 band (0.01-0.027 Hz)** |  |  |
| Right superior frontal gyrus, medial | Pearson correlation (*r*) | -0.046 |
|  | Significance (*p*) | 0.801 |
| Right middle frontal gyrus | Pearson correlation (*r*) | -0.238 |
|  | Significance (*p*) | 0.182 |
| Right middle frontal gyrus | Pearson correlation (*r*) | -0.083 |
|  | Significance (*p*) | 0.646 |
| Right middle frontal gyrus | Pearson correlation (*r*) | -0.112 |
|  | Significance (*p*) | 0.533 |

**Table S7. The corresponding regions of the cluster in other three functional atlas**

|  | **Coordinate (x, y, z)** | **Functional template** | | |
| --- | --- | --- | --- | --- |
|  |  | BASC | Schaefer | Gordon |
| **Conventional frequency band (0.01-0.08 Hz)** | | | | |
| **Cluster 1** | 9, 48, 24 | 133 | RH_DefaultB_PFCd_3 | Default |
| **Slow-5 band (****0.01-0.027 Hz)** | | | | |
| **Cluster 2** | 9, 45, 30 | 96 | RH_SalVentAttnB_PFCl_4 | Default |
| **Cluster 3** | 36, 57, 24 | 96 | RH_SalVentAttnB_PFCl_3 | DorsalAttn |
| **Cluster 4** | 48, 33, 36 | 175 | RH_ContB_PFCld_1 | FrontoParietal |
| **Cluster 5** | 39, 12, 51 | 175 | RH_ContB_PFCld_2 | FrontoParietal |
